# Social capital and its effect on business performance in the Nigeria informal sector

**DOI:** 10.1016/j.heliyon.2019.e02024

**Published:** 2019-07-25

**Authors:** Olamide Oluwabusola Akintimehin, Anthony Abiodun Eniola, Oluwatobi Joseph Alabi, Damilola Felix Eluyela, Wisdom Okere, Emmanuel Ozordi

**Affiliations:** a Department of Business Studies, Landmark University, Omu Aran, Nigeria; b Department of Sociology, University of Johannesburg, Johannesburg, South Africa; c Department of Accounting and Finance, Landmark University, Omu Aran, Nigeria; d Department of Economics, Accounting and Finance, Bells University of Technology, Ota, Nigeria; e Department of Accounting, Covenant University, Ota, Nigeria

**Keywords:** Business, Informal sector, Business performance, Social capital, Nigeria

## Abstract

This study was aimed at investigating the effect of internal and external social capital on the financial and non-financial performance of businesses in the Nigerian informal sector. The study further investigated the controlling role of firm age. A cross-sectional survey of 650 informal business owners in the Ikeja region of Lagos state, Nigeria was carried out. The analysis was carried out using the partial least square method of the structural equation model (SEM). Findings revealed that without the controlling variable of firm age, social capital had a significant effect on business performance, internal social capital had a significant effect on non-financial performance, it, however, had no significant effect on financial performance, while external social capital had no significant effect on financial and non-financial performance. With the controlling variable of firm age, social capital had a significant effect on business performance, internal social capital had a significant effect on financial and non-financial performance, while external social capital had no significant effect on financial and non-performance. The study, therefore, recommended that informal entrepreneurs take advantage of their internal social capital resources and also try to build their external social capital as they may become vital for their business success.

## 1 Introduction

The informal sector is popularly known as “grey economy” (Chima, 2016; Henshaw, 2017) plays a vital role in the economic growth of the Nigerian economy, contributing 41.43% to Nigeria's GDP (NBS, 2016), and 57.9% to Nigeria's GNP at US$212.6 billion (Schneider, 2002), and therefore still growing in terms of national GNP up till this present moment. Apart from this, the sector contributes to the mass reduction of the ever-growing unemployment rate in Nigeria (Asalaye et al., 2018a, Asalaye et al., 2018b) through the provision of employment opportunities to over 48 million Nigerians and also serving as a reliable waiting ground for Nigerian youths awaiting formal sector employment (Yusuf, 2014). The flexibility of the informal sector is reflected in its ability to accommodate those gainfully employed in the formal sector to create additional income avenues, popularly known as “side-hustle” in Nigeria. As a result of these, the relevance of the informal sector to the growth and development of the Nigerian economy cannot be overemphasized.

Despite the enormous contributions of the informal sector to the economic growth and development of Nigeria, it still faces a wide range of challenges from limited or no access to adequate resources (human and financial), technology, and market information needed for its growth and survival. Moreover, there seems not to be a development agenda for the informal sector in most African countries (Chima, 2016). As a result of these challenges, most businesses in the informal sector suffer instability and do not survive beyond three years (Agyapong et al., 2017). Nevertheless, business owners in the informal sector seem not to be able to create, expand and exploit the wealth of social capital at their disposal to ensure improved business performance and growth (Agyapong et al., 2017), even though research has shown that the amount of social capital rooted in an entrepreneurs' personal network is vital for the efficient performance of their business (Stam et al., 2014), it has not been properly conceptualized within Nigeria's business literature.

This social capital can as well be referred to as the social connections through which entrepreneurs can obtain tangible and intangible assets necessary for business performance (Dai et al., 2015). The resources informal entrepreneurs are able to access via their personal network and social connections enable them to identify business opportunities (Bhagavatula et al., 2010) and mobilize human and financial resources (Batjargal, 2003). Just as mentioned in the work of Dai et al. (2015), the operations of businesses in the informal sector tend to be influenced by both external and internal social capital. The external social capital enables credit support from creditors, the supply of valid market information and referrals from loyal customers while internal social capital enables support from family, friends and professional colleagues in terms of financial loans or gifts and strategic business advice.

Various studies have investigated the relationship between social capital and organizational performance (Felicio et al., 2014; Clarke et al., 2016; Pratono, 2018), alongside a meta-analytic review of 59 studies by Stam et al. (2014) on the effects of an entrepreneurs’ social capital on small firm performance. Also, most of the research done on social capital and firm performance have focused on developed economies like Europe and North America (Cantner and Stuetzer, 2010; Ndofor and Priem, 2011; Patel and Terjesen, 2011; Brink, 2011) and African countries like Ghana and Kenya (Agyapong et al., 2017; Bradley et al., 2012). However, search through literature has revealed that unpacking the relationship between entrepreneur social capital and the development of small-scale businesses have not been explored in Nigeria. A methodological gap was as well observed in previous studies. For instance, previous studies have adopted analytical tools such as meta-regression (Stam et al., 2014), ordinary least square regression (Agyapong et al., 2017), and hierarchical moderated regression analysis (Dai et al., 2015). Some other studies have as well adopted the structural equation modeling (SEM) tool (Pratono, 2018; Felicio et al., 2014). However, the SEM has never been adopted in any social capital related research in Nigeria. To this effect, this paper is aimed at filling the research gaps as identified.

This paper, therefore, investigated the effect of social capital resources on the business performance of the informal sector in Nigeria. According to Dai et al. (2015), it is important for researchers in this area to relatively investigate the contribution of external and internal social capital on the financial and non-financial performances of businesses in Nigeria's informal sector. This paper through an extensive review of literature and engagement of empirical findings has opened up conversions around the unstructured and informal avenues through which small businesses thrive in Nigeria. Through the exploration of social capital, it has un-hatched new narratives to small enterprise success and corroborated other findings in the literature that social capital influences the success of the small-scale business at its foundational stages.

## 2 Background

This section is aimed at discussing relevant concepts to this study (informal sector), as well as the theoretical framework underpinning the study. Lastly, a summary of empirical reviews was discussed.

### 2.1 Features of the informal sector

According to the International Conference of Labour Statisticians (1993), the informal sector comprises business units which carry out the production of goods and services with the aim of creating revenues and occupation to parties involved. The informal sector according to ILO (2002a,b; 2003) consists of wage workers including workers of informal enterprises, home workers, part-time employees, and remunerated domestic employees. World Bank (2003) also defines the informal sector as including employers in the form of proprietors and operators of informal business enterprises. The informal sector can be understood from the perspective of revenue and employment enhancing potential as; business enterprises with potentials to contribute to national economic growth and wealth creation, entities or families who engage in informal businesses for survival and individuals who commit to informal business activities alongside their formal jobs (Oberay and Chadaw, 2001; ILO (2002a,b).

The Nigerian informal sector is defined in terms of employee size as consisting of enterprises having less than ten (10) employees (Onyebueke and Geyer, 2011). The ownership structure of most informal enterprises comprises one-man sole proprietorship voluntarily assisted by family members and trainees popularly known as apprentices (Abumere et al., 1998; CBN/FOS/NISER, 2001, pp. 68–69; Oduh et al., 2008). This study adopts the definition of Onyebueke and Geyer (2011). Therefore, we define informal sector enterprises in terms of employee strength not more than ten (10).

### 2.2 Theoretical review

This section presents the theoretical framework related to the study and the relevance of this theory to the research.

#### 2.2.1 Social capital theory

In this paper, we employed the social capital theory which provides a useful theoretical lens in the study. Social capital springs from social network theory and our emphasis on social capital theory stem from the fact that social capital is embedded in relationships at many levels. The application of the social capital theory is aimed at comprehending social ties, social interaction, trust and reciprocity (Pratono, 2018).

Previous researches on social capital have suggested that it could be divided into tripartite dimensions (Anandi, 2000; Adler and Kwon, 2002; Aladwani, 2002; Chang et al., 2011 ). These are structural, relational and cognitive social capital. The structural dimension emphasizes the level of connection and closure amongst social network members, the relational dimension emphasizes the strength of these social relationships, and the key factors here are trust and trustworthiness, while the cognitive dimension emphasizes the shared values, norms and beliefs existing amongst the social network.

The social capital theory in all dimensions relates to this research in the sense that when an entrepreneur has a strong social network sharing the same values and beliefs, his business firm would be able to enjoy the various resources provided by his social network through a superior level of financial and non-financial performance.

### 2.3 Empirical reviews

Pratono (2018) investigated the mediating role of trust, selling capability and pricing capability on the relationship between social networks and firm performance using 380 small and medium enterprises (SMEs) in Indonesia as a study population, and the structural equation model (SEM) as a method of data analysis. Findings revealed that social networks enhance firm performance, with trust playing a key role in enabling social network structures to generate selling and pricing capabilities, thereby leading to a positive impact on firm performance.

Agyapong et al. (2017) investigated the mediating role of innovation on the relationship between social capital and micro & small businesses (MSBs) performance in an emerging economy using 500 MSBs in Ashanti, Ghana as a study population, and hierarchical regression analysis as a method of data analysis. Findings revealed that social capital directly influenced MSBs performance, and directly via innovation. It furthers reveals that innovation mediates the relationship between social capital and MSBs performance. Lastly, the study revealed the existence of a positive relationship between various types of innovation and MSBs performance.

Bachrach et al. (2016) conducted a matched survey of 246 sales personnel grouped within 54 teams in a North American Fortune-100 supplying firm, with the research aim of examining the effect of investing in team social capital (ITSC) on sales performance using path analysis, within the structural equation model (SEM) as a method of data analysis. Findings revealed an indirect positive relationship between ITSC and sales performance in the sense that, ITSC was positively related to team goal monitoring and learning effort, which increased commitment to service quality, thereby instigating sales performance.

Dai et al. (2015) examined the moderating role of entrepreneurial activities on the relationship between social capital (internal and external) and the financial performance of a sample of hotels in the Chinese hospitality industry using hierarchical moderated regression as a method of analysis. Their findings revealed that the collaboration of internal and external social capital has a positive effect on the firm's financial performance. Although this study more specifically revealed that external social capital was more positively linked with financial performance than internal social capital, suggesting that internal social capital is most likely going to influence non-financial performance than financial performance.

Stam et al. (2014) conducted a meta-analytic review of 61 business enterprises with the aim of examining the association between entrepreneurs’ social capital (personal networks) and small firm performance. Their findings primarily revealed the existence of a positively significant relationship between social capital and firm performance. Their findings further revealed the presence of moderating variables such as structural holes and network diversity, having larger positive effects on firm performance than personal networks. Their results as well revealed the dependence of social capital-firm performance relationship on firm age.

## 3 Hypothesis

**Fig. 1:**
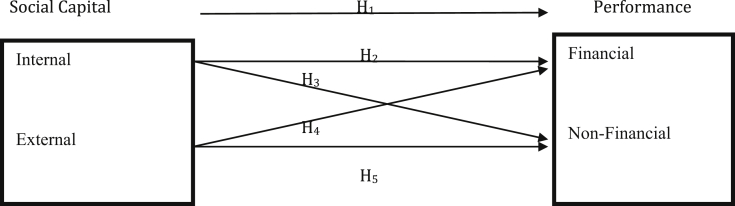
Conceptualization model.

**Source**: Adapted and modified from the works of Dai et al. (2015) and Agyapong et al. (2017).

This study aims at examining the effect of internal and external social capital on the financial and non-financial performance of informal businesses. Fig. 1 below shows the conceptualization model employed in this study, which was adapted from the works of Dai et al. (2015) and Agyapong et al. (2017).

### 3.1 Social capital and business performance

Social capital basically comprises the all existing and prospective resources available to an actor via his relationship network (Nahapiet and Ghostal, 1998). The social capital resources available to an entrepreneur tend to make all the difference in their business performance. Such that through social capital, easy access to intellectual, financial and cultural capital resource, which are very vital for efficient business performance, is ensured (Bourdieu, 1985). Apart from being able to provide easy access to these other vital capital resources, social capital can as well serve as a complement or substitute to them (Adler and Kwon, 2002). In serving as a substitute, entrepreneurs can compensate for their lack or limited access to financial and intellectual capital via superior network of relationships. As a complement, social capital can ensure financial efficiency and intellectual efficiency through the reduction of transaction costs (Lazerson, 1995) and knowledge sharing (Florin et al., 2003). As a result of these two vital roles social capital is able to play, it can be said to be an efficient capital in ensuring sustainable competitive advantage for a business.

However, social capital could be categorized as external social capital and internal social capital (Adler and Kwon, 2002). The external social is defined as the social connections with external bodies like loyal customers, suppliers, trade and professional associations, market authorities and creditors, as well as the intangible resources rooted in these connections such as trust, loyalty, and referrals. While internal social capital reveals social connections with friends, families, colleagues, business partners and employees, as well as the intangible resources rooted in these connections like trust, support, and strategic advice. These two categories of social capital are very vital to an entrepreneur such that, they are able to complement each other to ensure efficient business performance and sustainable competitive advantage.

Based on the above arguments, we propose the following hypothesis:

H1 Social Capital has a positive significant effect on business performance

### 3.2 Internal social capital and financial performance

The internal social network of an entrepreneur tends to affect the financial performance at the forming and introductory stage of his business through the ease and effectiveness in the mobilization of financial resources to start up the business initially, firstly because new businesses mostly start-ups with very limited amount of resources (Stam et al., 2014), and external potential resource providers may doubt the prospect of a new firm due to lack of legitimacy (Stinchcombe, 1965). In justification for this claim, financial support and no-interest loans from family, friends, colleagues or business associates tend to be one of the major sources of start-up capital for most informal businesses in Nigeria (Ibanga, 2015; Nguyen, 2015; Ibeleme, 2017). Such loans form a part of the internal social capital resources available to the informal entrepreneur.

There also tends to be dependence on these internal social networks for patronage and referrals leading to sales earnings at the introductory stage of the business which is usually characterized by low sales. They could as well provide linkage to suppliers in their own personal network, thereby ensuring cost reduction in raw materials acquisition which would lead to greater financial efficiency (Fafchamps and Minten, 2002).

Based on the above arguments, we propose the following hypothesis:

H2 Internal Social Capital has a positive significant effect on financial performance

### 3.3 Internal social capital and non-financial performance

Through interaction with internal social networks mostly comprising family and friends, informal entrepreneurs are able to enjoy easy access to market intelligence information and strategic advice which enables sustained competitive advantage. Internal social capital interactions also enable the easy accumulation of intellectual resources through knowledge sharing and mental collaborations which would enhance improved product or service quality (Florin et al., 2003).

Research has also suggested the adoption of social capital for family firms that aim to pursue innovation (Agyapong et al., 2017). Sanchez-Famoso et al. (2014) also added that the innovative capability of a firm depends on its ability to utilize the internal social capital resources at its disposal. This innovation which could be in form of product development, market development, and process control is highly essential for improved business performance in the informal sector because Nigerian customers highly price sensitive such that they always want to get the almost perfect value for their money in terms of high-quality products and services. Ability to meet their needs, therefore, requires a sufficient level of innovation. This innovation could, therefore, be achieved through the amassment of intellectual capital which is a vital resource available via internal social capital linkages and connections.

Based on the above arguments, we propose the following hypothesis:

H3 Internal Social Capital has a positive significant effect on non-financial performance

### 3.4 External social capital and financial performance

The existence of a strong positive relationship with external social networks of people relating to the business such as loyal customers, suppliers and professional associations/market authorities would have a positive impact on the sales revenue and profitability level of an informal business enterprise. In the case of customer loyalty, we find in the work of Jones and Taylor (2012) that these loyal customers are usually more than willing to pay more for a product or service and unlike many Nigerian consumers, they tend not to try to beat down the price given to them based on the existence of a relationship. Building strong and close links with market authorities and trade associations tend to facilitate access to valuable market information at a reduced cost (Landry, et al., 2002). Granovetter (1985, pp. 540) also adds that these lower price access to valuable market information is being compensated for by the effort made by an informal entrepreneur in building relationships with parties involved.

Finally, on the part of suppliers, just as identified in the work of Fafchamps and Minten (2002), financial efficiency is ensured via cost reduction in raw material acquisition, but this comes at the expense of building and maintaining close links with these suppliers.

Based on the above arguments, we propose the following hypothesis:

H4 External Social Capital has a positive significant effect on financial performance.

### 3.5 External social capital and non-financial performance

Through the establishment of a strong relationship with these external social networks whom could be in the form of loyal customers and suppliers, an informal business enterprise could enjoy a sustainable competitive advantage. Dai et al. (2015) noted that strong external relationships enable informal enterprises to obtain valuable market information from market authorities and trade/professional associations, thereby increasing the market share of such enterprise. Such valuable market information serves as external knowledge acquisition. This knowledge acquisition is a mechanism through which a business enterprise integrates ideas and expertise from its external business environment, into its existing knowledge base (Ortiz et al., 2017) thereby ensuring that it competes successfully (Liao and Marsillac, 2015). Strong customer relationships could lead to customer loyalty which is usually manifested by repurchase intentions, customer preference (Jones and Taylor, 2012) and sales referrals, thereby ensuring increased market share. Based on the above arguments, we propose the following hypothesis:

H5 External Social Capital has a positive significant effect on non-financial performance.

### 3.6 Methodology

The aim of this study was to investigate the effect of internal and external social capital resources on the financial and non-financial performance of enterprises in the informal sector. In achieving this, the cross-sectional survey design was adopted. This is because this research was interested in observing at only one point in time, the sample subjects without any attempt to manipulate them. While the selected survey method is quick, easy and inexpensive, it is not without its flaws, such that it cannot be used to analyse behaviour over a period of time in form of trends (Sedgwick, 2014).

The scope of this study was confined to the Lagos state, which is in the western region of Nigeria. The decision to use Lagos as our study population is justified by the following fact; as a state, it accounts for 30% of the economic activities in Nigeria, it has the largest collection of formal and informal employees in Nigeria, and it is the financial hub of Nigeria. However, the study is further delimited to the Ikeja Local Government Area of Lagos state. Ikeja was also selected because even in Lagos, most informal enterprises are domiciled and operated in the Ikeja region. Based on the homogenous nature of business enterprises which operate in the informal sector, a sample size of 650, considered large and representative enough, was selected for this study (Agyapong et al., 2017). A simple random sampling technique was adopted in the selection and collection of data from these 650 informal business owners.

Primary data was therefore collected through a survey of informal business owners who served as our research respondents. In the collection of these data, the self-administered questionnaire which was designed by the authors was used (For further details, please see the Appendix). Prior to this, a pilot test was carried out with 26 informal business owners so as to test the degree at which the questionnaire is accurate, some minor adjustments were made to the questions based on this pilot survey. The main survey was conducted between 21^st^ October and December 2^nd^, 2018 with the aid of two research assistant personnel. During the data collection process, 643 questionnaires were returned, resulting in a 99% response rate, but out of them, 43 questionnaires were either incorrectly filled or uncompleted, therefore they were discarded. As a result, our final sample size for data analysis was 600. Cronbach Alpha and composite reliability were used in testing for the reliability of the chosen instrument, descriptive statistical analysis was done using SPSS version 23 and our research data was analysed via hypothesis testing using structural equation modeling (SEM-PLS) partial least square method version 3.2 (Pratono, 2018).

In collecting and analysing the research data, ethical approval was granted for the adoption of our research instrument by the Landmark University ethical committee.

### 3.7 Measurement of constructs

This section entails the discussion of major constructs adopted in this research: Firm Performance and Social Capital.

#### 3.7.1 Performance

This construct was measured on the basis of financial and non-financial performance. Indicators of the financial performance consisted of revenue earnings, market share growth, returns on investment, cost efficiency and overall financial performance. (Eluyela et al., 2018a, Eluyela et al., 2018b). While the indicators of non-financial performance consist of product quality, customer satisfaction, customer preference, customer loyalty, customer service, product/service innovation, patronage, competitive position, and market size. In measuring these financial and non-financial performance variables, subjective measures were adopted such that respondents were asked to compare their financial and non-financial performance with that of their competitors on a 5-point scale (1 = much worse than competitors; 5 = much better than competitors). The decision to adopt subjective measures when assessing financial and non-financial performance is due to findings that subjective measures are more easily reached than objective measures, thereby making them very vital, reliable and valid (Richard et al., 2010, Sciascia and Mazzola, 2008, Dess and Robinson, 1984).

#### 3.7.2 Social capital

Social capital was measured on the basis of internal and external social capital resources. Indicators of internal social capital resources consisted easy access to business advice, knowledge sharing, patronage and referrals from the internal social networks of people available to an entrepreneur, comprising majorly of family and friends, as well as employees/apprentices. In measuring internal social capital, family, friends, employees/apprentices were adopted. While the indicators of external social capital resources consisted strong rapport with loyal customers, suppliers, market authorities and other relevant authorities leading to brand loyalty, referrals, access to valuable market information at little or no cost and cost reduction in raw material acquisition. However, it is important to note that the sub-variables (customers, suppliers and market authorities) chosen to measure external social capital are just subcomponents of the total external social capital (Westlund, 2006, pp. 52; Westlund and Nilsson, 2005). Subjective measures were also adopted to measure the extent to which the respondents enjoyed the existence of internal and external social capital resources in their respective business firms on a 5-point scale (1 = strongly disagree; 5 = strongly agree).

#### 3.7.3 Control variables

This study controlled for firm age alone as proxy by duration of firm existence as a non-perceptual measure (Atinc et al., 2012) to reduce error terms and increase statistical power (Becker, 2005). Moreover, the works of O'Neill, (2004); O’Neill and Xiao, (2006); and Xiao et al. (2012), suggested that performance could be influenced by a firm's age. In controlling for firm age, this research took into account the duration of firm existence (Dai et al., 2015).

## 4 Results and discussion

The study conducted a descriptive statistical analysis to look into the overall mean and standard deviation, so as to determine the distributions of the coefficient of variation (see Table 1).

**Table 1:** Overall descriptive statistical table.

Statistics							
		Duration Of Firm Existence	Ownership Structure	Ownership Type	Firm Industry	Business Engagement Level	Firm Size
N	Valid	600	600	600	600	600	600
	Missing	0	0	0	0	0	0
Mean		2.5017	1.2883	1.7933	3.1367	1.2217	1.5733
Std. Deviation		1.23391	.50888	.40525	1.08009	.41571	.64185
Minimum		1.00	1.00	1.00	1.00	1.00	1.00
Maximum		5.00	3.00	2.00	4.00	2.00	4.00

**Source**: Field survey (2019).

The overall mean values indicate the firm industry and duration of firm existence in terms of firm age are with the highest mean value among the alternatives under examination. It is an indication that the types of firm industry in the society coupled with the firm age exercise the greatest degree of influence on business performance with the highest mean value of 3.14 and 2.50 respectively. Likewise, the standard deviations are greater than one; which is an indication that there is a relatively high variation among the respondents as to the influence and effect of firm age and the types of industry on business performance. However, ownership structure, ownership types, business engagement level, and firm size have distributions with a coefficient of variation lower than 1 are considered being low-variance with the distributions.

The results in Table 2 show that 147 (24.5%) firms had existed for a duration of 1–2 years, 178 (29.7%) firms had existed for a duration of 3–4years, 163 (27.2%) firms had existed for a duration of 5–6 years, 51 (8.5%) firms had existed for a duration of 7–8years, and 61 (10.2%) firms had existed for a duration of more than 8years.

**Table 2:** Duration of firm existence.

		Frequency	Percent	Valid Percent	Cumulative Percent
Valid	1–2years	147	24.5	24.5	24.5
	3–4years	178	29.7	29.7	54.2
	5–6years	163	27.2	27.2	81.3
	7–8years	51	8.5	8.5	89.8
	above 8years	61	10.2	10.2	100.0
	Total	600	100.0	100.0	

**Source**: Field survey (2019).

The results in Table 3 show that the ownership structure operated by 443 (73.8%) firms was sole proprietorship ownership structure, the ownership structure of 141 (23.5%) was partnership, and 16 (2.7%) firms had ownership structures other than sole proprietorship and partnership.

**Table 3:** Ownership structure.

		Frequency	Percent	Valid Percent	Cumulative Percent
Valid	sole proprietorship	443	73.8	73.8	73.8
	Partnership	141	23.5	23.5	97.3
	Others	16	2.7	2.7	100.0
	Total	600	100.0	100.0	

**Source**: Field survey (2019).

The results in Table 4 show that 124 (20.7%) firms were family-owned, while 476 (79.3%) were non-family owned.

**Table 4:** Ownership type.

		Frequency	Percent	Valid Percent	Cumulative Percent
Valid	family-owned	124	20.7	20.7	20.7
	nonfamily-owned	476	79.3	79.3	100.0
	Total	600	100.0	100.0	

**Source**: Field survey (2019).

The result in Table 5 shows that 78 (13%) firms were in the manufacturing sector, 80 (13.3%) firms were in the retail sector, 124 (20.7%) firms were in the textile sector, and 318 (53%) were in the service sector.

**Table 5:** Firm industry.

		Frequency	Percent	Valid Percent	Cumulative Percent
Valid	manufacturing	78	13.0	13.0	13.0
	Retail	80	13.3	13.3	26.3
	Textile	124	20.7	20.7	47.0
	Service	318	53.0	53.0	100.0
	Total	600	100.0	100.0	

**Source**: Field survey (2019).

The result in Table 6 shows that 467 (77.8%) firms were operated on a full-time basis, while 133 (22.2%) were operated on a part-time basis.

**Table 6:** Business engagement level.

		Frequency	Percent	Valid Percent	Cumulative Percent
Valid	full-time engagement	467	77.8	77.8	77.8
	part-time engagement	133	22.2	22.2	100.0
	Total	600	100.0	100.0	

**Source**: Field survey (2019).

The result in Table 7 shows that 303 (50.5%) firms had 0-3 employees/apprentices, 253 (42.2%) firms had 4-7 employees/apprentices, 41 (6.8%) firms had 8-11 employees/apprentices, while 3 (0.5%) firms had above 11 employees/apprentices.

**Table 7:** Firm size.

		Frequency	Percent	Valid Percent	Cumulative Percent
Valid	0-3 employees/apprentices	303	50.5	50.5	50.5
	4-7 employees/apprentices	253	42.2	42.2	92.7
	8-11employees/apprentices	41	6.8	6.8	99.5
	above 11 employees/apprentices	3	.5	.5	100.0
	Total	600	100.0	100.0	

**Source**: Field survey (2019).

### 4.1 Measurement model assessment

Structural Equation Modeling (SEM) is used on the theoretical framework. Partial Least Square (PLS) method can handle many independent variables, even when multicollinearity exists. PLS can be implemented as a regression model, predicting one or more dependent variables from a set of one or more independent variables or it can be implemented as a path model. Partial Least Square (PLS) method can associate with the set of independent variables to multiple dependent variables (Khoi and Van Tuan, 2018; Latan and Noonan, 2017; Wong, 2013).

**Fig. 2:**
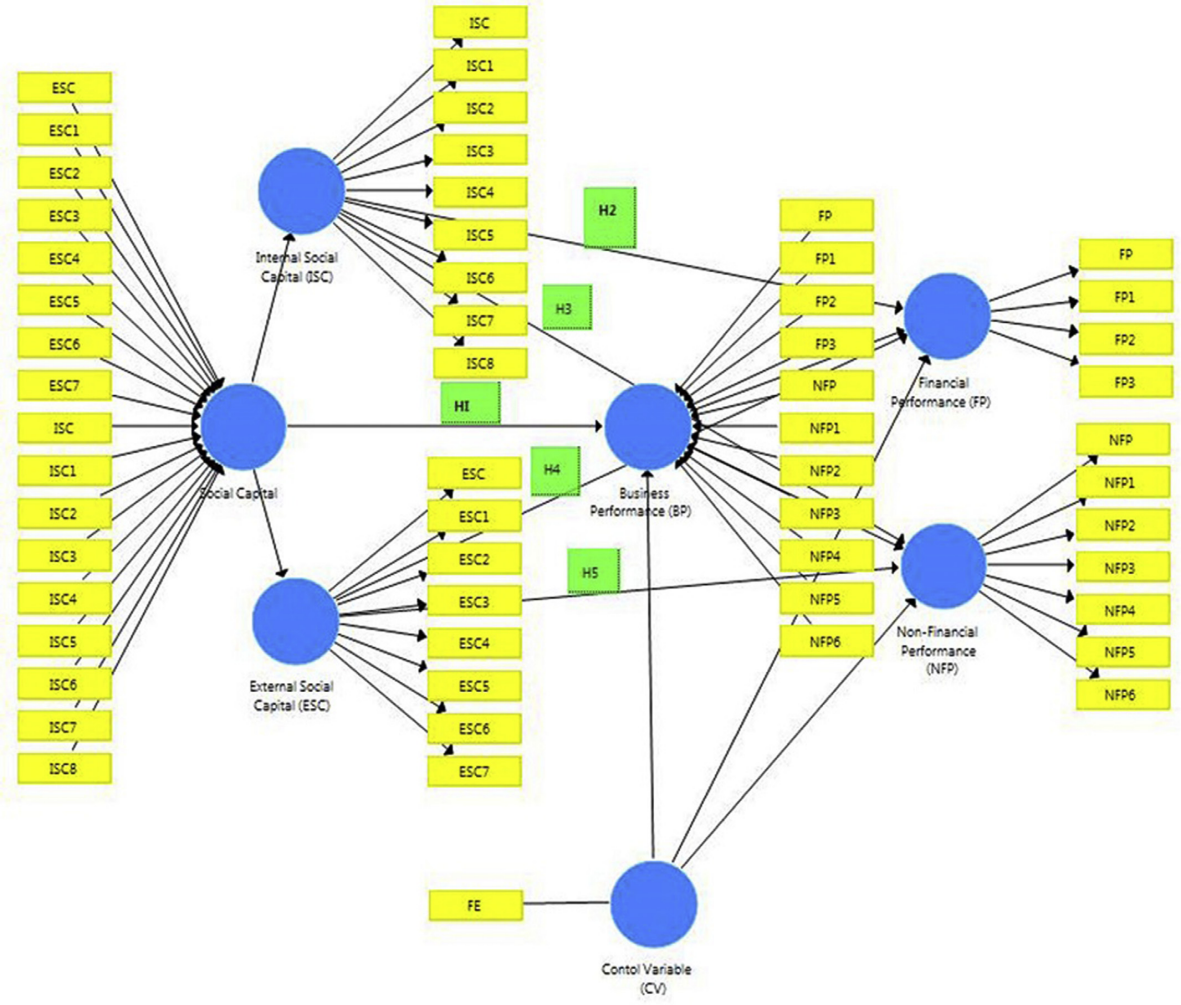
Conceptual model.

Source: Author's Computation (2019)

Fig. 2 above is based on the conceptual framework that has been designed earlier in this paper (see Fig. 1), an inner and outer model was therefore developed in SmartPLS showing the path-coefficient between social capital (SC) and business performance (BP) without the control variable of firm age (duration of firm existence-FE). The study path coefficients which would be developed later in this paper would show the strength of the relationships amongst all variables of social capital (internal social capital- ISC and external social capital-ESC) and business performance (financial performance-FP and non-financial performance-NFP), to verify whether these relationships are statistically significant.

The labels ISC1-8, ESC1-6, FP1-3 and NFP1-6 signify each construct representing internal social capital (ISC), external social capital (ESC), financial performance (FP) and non-financial performance (NFP) as shown in the questionnaire questions in each section of these constructs (ISC, ESC, FP, NFP) (see appendix).

### 4.2 Indicator reliability

This represents how much of the variation in an item is explained by a variable (Taylor and Geldenhuys, 2019; Asgari, 2016; Hair et al., 2013b). Indicator reliability was assessed using the outer loadings as shown below. Higher outer loading on a variable indicates that the associated measure has much in common, that is measured by the variable (Taylor and Geldenhuys, 2019; Hair et al., 2013b; Wong, 2013). Memon and Rahman (2014) and Hair et al., 2013b also suggested that items having a loading >0.70 should be retained, items having an outer loading value > 0.40 should be omitted and that its impact on the average variance extracted (AVE) and composite reliability (CR) of the variable should be analyzed. If the AVE and CR of the variable reach above the threshold value, then the given item should be omitted; otherwise, it should be retained. Seven reflective measures from sub-constructs of social capital (SC), two from internal social capital (ISC) along with one reflective indicator from business performance (BP) were omitted. Omitting these items resulted in an increase in CR and AVE above the suggested threshold values of 0.70 and 0.50, respectively (Hair et al., 2013b). We next provide the assessment of our structural model.

### 4.3 Measurement model assessment

In PLS-SEM, assessment of the measurement model (also referred to as the outer model) includes CR to evaluate internal consistency, individual indicator reliability, and AVE to evaluate convergent validity (Hair et al., 2013b).

### 4.4 Consistency and reliability

As indicated in Table 8, to access the convergent validity, analysis of the factors loading, of the CR and the AVE were simultaneously used. The convergent validity indicated that all factors loading were significant at 0.01. In this reflective model, convergent validity was tested through composite reliability or Cronbach's alpha. Composite reliability (CR) is the measure of reliability since Cronbach's alpha sometimes underestimates the scale reliability (Khoi and Van Tuan, 2018; Latan and Noonan, 2017; Wong, 2013).

**Table 8:** Factor loading.

Loading	Business Performance (BP)	Financial Performance (FP)	Non-Financial Performance (NFP)	Social Capital (SC)	External Social Capital (ESC)	Internal Social Capital (ISC)
FP	0.399	0.525				
FP1	0.786	0.859				
FP2	0.786	0.867				
FP3	0.790	0.828				
NFP	0.723		0.744			
NFP1	0.716		0.779			
NFP2	0.687		0.761			
NFP3	0.715		0.781			
NFP4	0.734		0.800			
NFP5	0.731		0.780			
NFP6	0.725		0.757			
ESC				0.534	0.658	
ESC1				0.589	0.672	
ESC2				0.585	0.729	
ESC3				0.581	0.712	
ESC4				0.507	0.638	
ESC5				0.462	0.556	
ESC6				0.537	0.608	
ESC7				0.455	0.534	
ISC				−0.245		
ISC1				−0.221		
ISC2				0.234		0.359
ISC3				0.572		0.742
ISC4				0.579		0.760
ISC5				0.616		0.803
ISC6				0.565		0.766
ISC7				0.425		0.663
ISC8				−0.053		

Source: Author's Computation (2019)

According to Henseler et al. (2009), R^2^ values of .67, .33, and .19 are substantial, moderate, and weak respectively. The coefficient of determination results reflects that FP and NFP R^2^ is substantial while R^2^ BP is moderate in the usefulness of a regression model as shown in Table 2.

Table 9 shows that CR varied from 0.847 to 0.911 which was above the preferred value of 0.5. This proves that the model is internally consistent. All constructs factor loading exceeded the 0.50 cut-off, with the exception of (ESC) External Social capital (AVE = 0.412). However, the ESC dimensions were found to have adequate convergent validity based on their high composite reliability (>0.70) (Gerbing and Anderson, 1988).

**Table 9:** Construct reliability and validity.

Variables	No of Items	Cronbach's Alpha	R Square	Composite Reliability	Average Variance Extracted (AVE)
External Social Capital (ESC)	8	0.794		0.847	0.412
Internal Social Capital (ISC)	5	0.807		0.866	0.564
Financial Performance (FP)	3	0.829	0.829	0.898	0.746
Non-Financial Performance (NFP)	7	0.887	0.870	0.911	0.595
Business Performance (FP)			0.290		

Source: Author's Computation (2019)

**Table 10:** Correlations and measures of validity among variables.

Variables	AVE	External Social Capital	Financial Performance	Internal Social Capital	Non-Financial Performance
External Social Capital (ESC)	0.412	0.642			
Financial Performance (FP)	0.746	0.429	0.864		
Internal Social Capital (ISC)	0.564	0.250	0.266	0.751	
Non-Financial Performance (NFP)	0.595	0.444	0.707	0.367	0.771

**Table 11:** Assessment of Formative model.

Collinearity Statistics (VIF)	
Outer VIF Values	
ESC	1.851
ESC1	1.917
ESC2	1.788
ESC3	1.652
ESC4	1.454
ESC5	1.351
ESC6	1.634
ESC7	1.499
FP1	1.955
FP2	2.111
FP3	1.741
ISC3	1.629
ISC4	1.740
ISC5	1.844
ISC6	1.769
ISC7	1.527
NFP	2.153
NFP1	2.525
NFP2	1.939
NFP3	2.019
NFP4	2.223
NFP5	2.330
NFP6	2.031

**Table 12:** Path coefficients for hypothesis analysis.

	Original Sample (O)	Sample Mean (M)	Standard Deviation (STDEV)	T Statistics (|O/STDEV|)	P Values
Social Capital - > Business Performance (BP) (H1)	0.539	0.554	0.032	16.802	0.000
Internal Social Capital (ISC) - > Financial Performance (FP) (H2)	−0.055	−0.055	0.029	1.929	0.054
Internal Social Capital (ISC) - > Non-Financial Performance (NFP) (H3)	0.048	0.042	0.023	2.031	0.043
External Social Capital (ESC) - > Financial Performance (FP)_(H4)	−0.009	−0.009	0.023	0.397	0.692
External Social Capital (ESC) - > Non-Financial Performance (NFP) (H5)	−0.006	−0.011	0.021	0.266	0.791

***: p<0.001; **: p < 0.01; *: p < 0.05.

**Table 13:** Path Coefficients for Hypothesis Analysis with a control variable.

	Original Sample (O)	Sample Mean (M)	Standard Deviation (STDEV)	T Statistics (|O/STDEV|)	P Values
Social Capital - > Business Performance (BP) (H1)	0.514	0.529	0.030	16.874	0.000
Internal Social Capital (ISC) - > Financial Performance (FP) (H2)	−0.056	−0.055	0.028	2.019	0.044
Internal Social Capital (ISC) - > Non-Financial Performance (NFP) (H3)	0.044	0.038	0.022	1.990	0.047
External Social Capital (ESC) - > Financial Performance (FP)_(H4)	−0.008	−0.007	0.025	0.336	0.737
External Social Capital (ESC) - > Non-Financial Performance (NFP) (H5)	−0.004	−0.010	0.022	0.185	0.853

***: p < 0.001; **: p < 0.01; *: p < 0.05.

### 4.5 Discriminant validity

To support the construct validity of the outer model, it was necessary to establish the discriminant validity. Discriminant validity reflects the extent to which the measure is unique and not simply a reflection of other variables (Peter and Churchill, 1986). The criterion and cross-loading scores of Fornell and Larcker (1981) were used to establish discriminant validity. Table 3 demonstrates that the square roots of AVE for all latent variables were higher than the inter-construct correlations (Fornell and Larcker, 1981) and therefore, they confirm discriminant validity.

Further, all indicators of individual loadings were found to be higher than their respective cross-loadings (Hair et al., 2013b). This provides additional evidence for discriminant validity (see Tables 10–13).

### 4.6 Assessment of formative model

Performance is a formative construct in the PLS path model and it is assessed for validity for Collinearity issue and significance and relevance of indicators (by checking outer weights and outer loadings) (Eniola, 2018; Eniola and Entebang, 2017). All indicators have a VIF value <5 as shown in Table 4. Hence, there is no collinearity issue present between the indicators.

### 4.7 Regression analysis

**Fig. 3:**
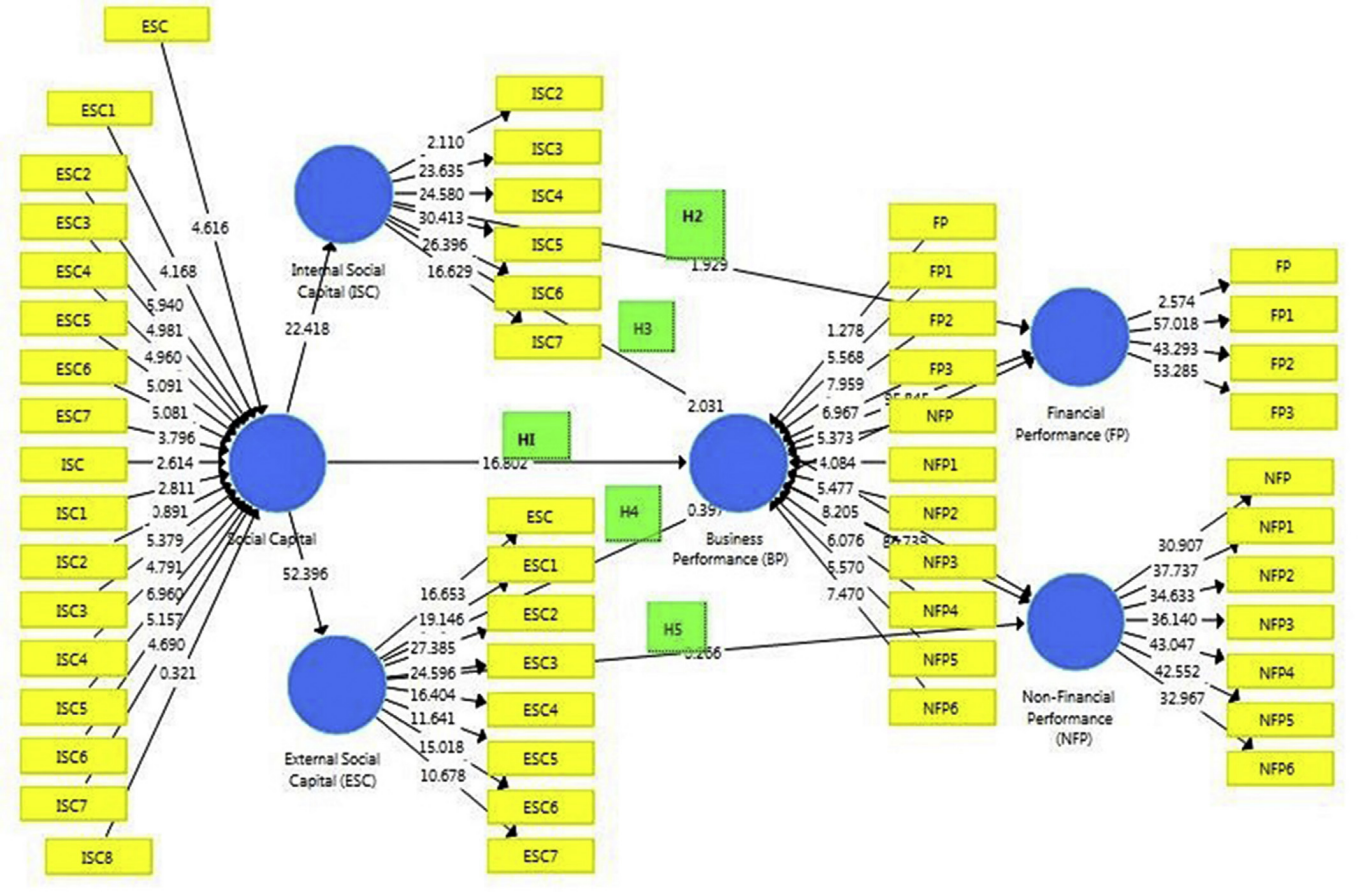
Path-Coefficient between social capital (SC) and business performance (BP) without the control variable.

Source: Author's Computation (2019)

**Fig. 4:**
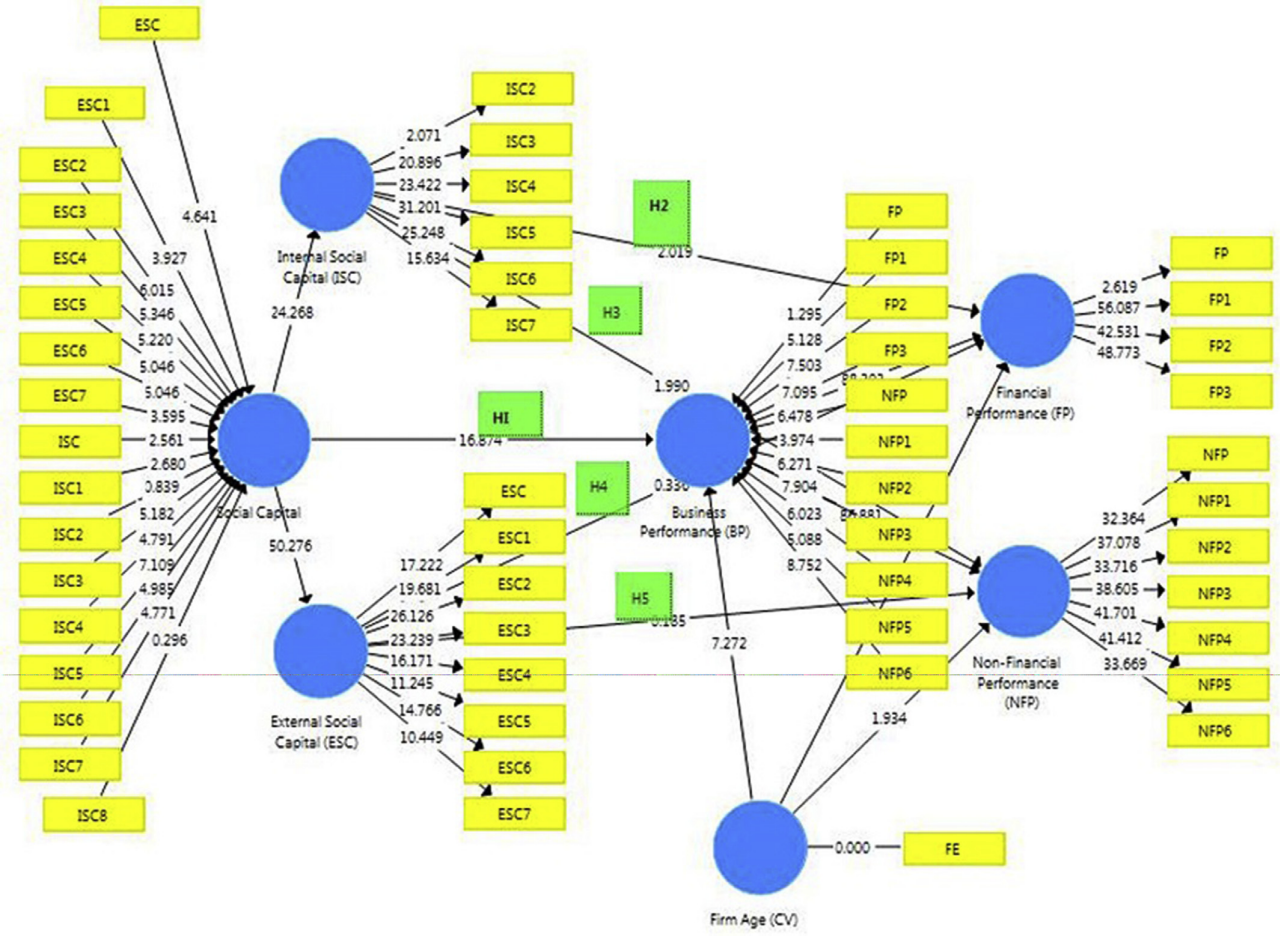
Path-Coefficient between SC and BP with a control variable.

Source: Author's Computation (2019)

Figs. 3 and 4, shows the path coefficient for the direct relationship between SC and other constructs. Nonparametric bootstrapping routine advocated by Vinzi et al. (2010), has been used on 122 data points and 5000 samples. “Bootstrapping is a re-sampling approach that draws random samples (with replacements) from the data and uses these samples to estimate the path model multiple times under slightly changed data constellations” (Hair et al., 2013a, pp. 162). The main purpose of bootstrapping is to calculate the standard error of coefficient estimates in order to examine the coefficient's statistical significance (Vinzi et al., 2010).

As discussed earlier, Fig. 3 above is based on the conceptual framework that has been designed earlier in this paper (see Fig. 1 and Fig. 2). An inner and outer model was therefore developed in SmartPLS showing the path-coefficient between social capital (SC) and business performance (BP) without the control variable of firm age. The study path coefficients, therefore, denoted the strength of the relationships amongst all variables of social capital (internal social capital- ISC and external social capital-ESC) and business performance (financial performance-FP and non-financial performance-NFP), to verify whether these relationships are statistically significant.

### 4.8 The assessment of the hypotheses testing procedures

As shown in Table 6 to test the hypothesis, Running the PLS algorithm and Bootstrapping calculations in SmartPLS software provided the path coefficient of these relations which denotes the strength of the relationships and P value for verifying whether the relationship is statistically significant.

From the table above, results show the following; Existence of a positively significant relationship between social capital and business performance (with a p-value of <0.01), thus providing support for earlier stated hypothesis (H1); Existence of a positively insignificant relationship between internal social capital and financial performance (with a p-value of 0.054; >0.05), thus not providing support for H2; Existence of a positively significant relationship between internal social capital and non-financial performance (with a p-value of 0.043; <0.05), thus providing support for H3; Existence of a positively insignificant relationship between external social capital and financial performance (with a p-value of 0.692; >0.05), thus not providing empirical support for H4; Existence of a positively insignificant relationship between external social capital and non-financial performance (with a p-value of 0.791; >0.05), thus not providing empirical support for H5.

As discussed earlier, Fig. 4 above is based on the conceptual framework that has been designed earlier in this paper (see Fig. 1–3). An inner and outer model was therefore developed in SmartPLS showing the path-coefficient between social capital (SC) and business performance (BP) with the control variable of firm age. The study path coefficients, therefore, denoted the strength of the relationships amongst all variables of social capital (internal social capital- ISC and external social capital-ESC), business performance (financial performance-FP and non-financial performance-NFP) and firm age (FE-firm existence), to verify whether these relationships are statistically significant.

From the table above, the results are a little different from that of the first analysis which was done without the control variable of firm age, although consistent with findings from previous analysis such as; the existence of a positively significant relationship between social capital and business performance (with a p-value of <0.01); the existence of a positively significant relationship between internal social capital and non-financial performance (with a p-value of 0.047; <0.05) the existence of a positively insignificant relationship between external social capital and financial performance (with a p-value of 0.737; >0.05); and the existence of a positively insignificant relationship between external social capital and non-financial performance (with a p-value of 0.853; >0.05). The only alteration in this finding revealed the presence of a positively significant relationship between internal social capital and financial performance (with a p-value of 0.044; <0.05), thus providing support for H_2_.

### 4.9 Theoretical findings

The findings of this work relate to the social capital theory. From the structural, relational and cognitive perspectives of this theory, it was discovered in this research that Nigerian informal entrepreneurs possess stronger connections with their internal social networks in which they share the same values, norms, and beliefs validated by trustworthiness, unlike their external social networks validated by less trustworthiness and emotional attachments, but more legitimacy (Stam et al., 2014; Hite and Hesterly, 2001; Stinchcombe, 1965). This legitimacy is, however, not enjoyed by most (81.3%) of the Nigerian informal enterprises investigated in this research because they were in existence between 1-6 years ago, and this period represents the formative/introductory stage of an informal business enterprise.

## 5 Discussion and conclusions

This study contributes to existing research on the subject of social capital by investigating the effects of internal and external social capital on the financial and non-financial performances of firms in the Nigerian informal sector. Prior to this study, various studies had investigated the effect of social capital on business performance as a whole without stated emphasis on internal or external social capital, financial or non-financial performance (Stam et al., 2014), some studies investigated the effect of internal and external social capital on only financial performance (Dai et al., 2015), effect of only internal social capital on sales performance (Bachrach et al., 2016), effect of only internal social capital on non-financial performance (Madhavaram and Hunt, 2017; Mani and Lakhal, 2015), effect of only external social capital on financial performance (Zhang and Fung, 2006), and effect of only external social capital on non-financial performance (Chang et al., 2011). To the best of the researcher's knowledge, no research had investigated the effect of both internal and external social capital on the financial and non-financial performance of a firm in the informal sector as a whole. Various studies had also had also focused on developed economies like Europe and North America (Cantner and Stuetzer, 2010; Ndofor and Priem, 2011; Patel and Terjesen, 2011; Patel and Terjesen, 2011; Brink, 2011) and African countries like Ghana and Kenya (Agyapong et al., 2017; Bradley et al., 2012), but none in Nigeria till date.

This study contributed to existing knowledge by picking up on the suggestions for further research in the work of Dai et al. (2015), recommending that further studies should comparatively examine the effect of internal and external social capital on firm financial and non-financial performance. And lastly, this research contributed by involving the control variable of firm age in influencing the effect of internal and external social capital on firm financial and non-financial performance.

### 5.1 Research limitations and suggestions for further studies

While conducting this research, some limitations were encountered. The study was delimited to the Lagos informal sector and used to represent the entire Nigeria informal sector. Future research should geographically cover a wider region in terms of scope for better representativeness, comparative analyses of informal sectors amongst informal economies in Africa or other continents could be carried out.

Lastly, future researches should investigate the impact of internal and external social capital on financial and non-financial performance in other sectors of the economy such as the hospitality and service sector.

### 5.2 Conclusion and recommendations

This research investigates the effect of social capital on firm performance in the Nigerian informal sector. The research also aims to comparatively investigate the following with and without involving the control variable of firm age;

a. The effect of internal social capital on firm financial performance
b. The effect of internal social capital on firm non-financial performance
c. The effect of external social capital on firm financial performance
d. The effect of external social capital on firm non-financial performance

**Without involving the control variable of firm age, the following findings revealed thus;**

a. Social capital has a significant effect on firm performance:
  *This finding is consistent with the works of* *Agyapong et al. (2017)* *and* *Stam et al. (2014)**.*
b. Internal social capital has no significant effect on firm financial performance:
  *This finding is consistent with the work of* *Dai et al. (2015)* *who suggested that internal social capital may likely have a positive significant effect on non-financial performance instead, but this finding is inconsistent with the work of* *Bachrach et al. (2016)**.*
c. Internal social capital has a significant effect on firm non-financial performance
  *This finding is consistent with the work of* *Bachrach et al. (2016)**,* *Madhavaram and Hunt, (2017)**,* *Mani and Lakhal, (2015)**, and the suggestion of* *Dai et al. (2015)* *as earlier stated.*
d. External social capital has no significant effect on firm financial performance:
  *This finding is inconsistent with the work of* *Dai et al. (2015)* *and* *Zhang and Fung, (2006)**.*
e. External social capital has no significant effect on firm non-financial performance:
  *This finding is inconsistent with the work of* *Chang et al., 2011* *.*

**While involving the control variable of firm age, the following findings revealed thus;**

a. Social capital has a significant effect on firm performance
b. Internal social capital has a significant effect on firm financial performance

*This finding is consistent with the work of**Mani and Lakhal, (2015)**,**Bachrach et al., (2016)**and**Sanchez-Famoso et al. (2014)**. Nevertheless, internal social capital had no significant effect on firm financial performance when the control variable of firm age was not involved, this shows a discrepancy. This discrepancy, therefore, reveals that this significance is majorly dependent on firm age. This dependence is as a result of the fact that from the descriptive statistics, 81.3% of the firms in this research were in existence between 1-6 years ago, and this period represents the formative/introductory stage of an informal business enterprise. Thus this finding shows consistency with earlier review of literature (**Stam et al., 2014**;**Stinchcombe, 1965**) which claims that internal social capital in form of mostly family, friends and employees/apprentices tend to enhance the financial performance of a firm via offering of abundantfinancial supportand contributions, as well as interest-free loans whenever needed, mostly at the introductory stages of these firms, in which external financial resource providers who form part of the external social capital would be reluctant to offer any kind offinancial supportassistance since the business lacks legitimacy at its formative stage.*

c. Internal social capital has a significant effect on firm non-financial performance
d. External social capital has no significant effect on firm financial performance
e. External social capital has no significant effect on firm non-financial performance

Our findings in the context of the Nigerian informal sector emphasizes the need for entrepreneurs to pay close attention to its internal social capital as they appeared to have significant effects on financial and non-financial aspects of business performance. Despite the fact that external social capital appeared to have a non-significant effect on both financial and non-financial performance due to the peculiarity of the Nigerian informal sector, they are as well important, there is a possibility of them being more important than internal social capital as their businesses become more established and mature, because by then, they would have superior legitimacy, thereby making it easier to enjoy financial and non-financial resources from external social capital sources (Hite and Hesterly, 2001) without any unnecessary emotional attachments.

Nevertheless, it is important for entrepreneurs to build on their existing internal social capital especially in periods where their respective businesses are still in the formative stage. It is also recommended for informal entrepreneurs to maintain quality relationships with their families, friends, and colleagues, and to ensure absolute trust and social cohesion between themselves and their respective business partners, employees or apprentices.

## Declarations

### Author contribution statement

Olamide Oluwabusola Akintimehin: Conceived and designed the experiments; Performed the experiments; Analyzed and interpreted the data; Contributed reagents, materials, analysis tools or data; Wrote the paper.

Anthony Abiodun Eniola: Performed the experiments; Analyzed and interpreted the data; Contributed reagents, materials, analysis tools or data; Wrote the paper.

Oluwatobi Joseph Alabi: Conceived and designed the experiments; Performed the experiments; Contributed reagents, materials, analysis tools or data; Wrote the paper.

Damilola Felix Eluyela, Wisdom Okere, Emmanuel Ozordi: Performed the experiments; Contributed reagents, materials, analysis tools or data; Wrote the paper.

### Funding statement

This research did not receive any specific grant from funding agencies in the public, commercial, or not-for-profit sectors.

### Competing interest statement

The authors declare no conflict of interest.

### Additional information

No additional information is available for this paper.

## Appendix ASupplementary data

Appendix_V2
